# Factors associated with a higher rate of distant failure after primary treatment for glioblastoma

**DOI:** 10.1007/s11060-013-1279-z

**Published:** 2013-10-18

**Authors:** Sonia Tejada, Ricardo Díez-Valle, Guillermo Aldave, Miguel Marigil, Jaime de Gallego, Pablo Daniel Domínguez

**Affiliations:** 1 Department of Neurosurgery, Clínica Universidad de Navarra, C/Pio XII, 36, 31008 Pamplona, Spain; 2 Department of Neurology, Clínica Universidad de Navarra, C/Pio XII, 36, 31008 Pamplona, Spain; 3 Department of Radiology, Clínica Universidad de Navarra, C/Pio XII, 36, 31008 Pamplona, Spain

**Keywords:** Pattern of recurrence, Glioblastoma, Fluorescence guided surgery, Tumor volume

## Abstract

Our purpose was to analyze the pattern of failure in glioblastoma (GBM) patients at first recurrence after radiotherapy and temozolomide and its relationship with different factors. From 77 consecutive GBM patients treated at our institution with fluorescence guided surgery and standard radiochemotherapy, 58 first recurrences were identified and included in a retrospective review. Clinical data including age, Karnofsky performance score, preoperative tumor volume and location, extend of resection, MGMT promoter methylation status, time to progression (PFS), overall survival (OS) and adjuvant therapies were reviewed for every patient. Recurrent tumor location respect the original lesion was the end point of the study. The recurrence pattern was local only in 65.5 % of patients and non-local in 34.5 %. The univariate and multivariate analysis showed that greater preoperative tumor volume in T1 gadolinium enhanced sequences, was the only variable with statistical signification (*p* < 0.001) for increased rate of non-local recurrences, although patients with MGMT methylation and complete resection of enhancing tumor presented non-local recurrences more frequently. PFS was longer in patients with non-local recurrences (13.8 vs. 6.4 months; *p* = 0.019, log-rank). However, OS was not significantly different in both groups (24.0 non-local vs. 19.3 local; *p* = 0.9). Rate of non-local recurrences in our series of patients treated with fluorescence guided surgery and standard radiochemotherapy was higher than previously published in GBM, especially in patients with longer PFS. Greater preoperative enhancing tumor volume was associated with increased rate of non-local recurrences.

## Introduction

Glioblastoma (GBM) is the most frequent malignant primary brain tumor in adults. Despite recent advances on treatment the prognosis of GBM patients is still dismal, with overall survival (OS) less than 10 % at 5 years [1]. Although autopsy studies suggest that GBM is diffusely disseminated within the brain most of the recurrences occur locally, i.e. at the primary site of the contrast enhancing lesion. Thus, rates of local recurrence of 80–100 % are widely accepted [2, 3].

The standard of care for GBM has changed during the past decade with the addition of concomitant and adjuvant chemotherapy with temozolomide (TMZ) to radiotherapy [4] Also surgical approach has changed, with both fluorescence guided surgery (FGS) with 5-aminolevulinic acid (ALA) [5–7], and intraoperative magnetic resonance imaging (MRI) [8, 9] leading to rates of complete resection of the enhancing tumor (CRET) much greater than previously reported [10].

Most groups that have reviewed the issue in the present decade agree that the addition of TMZ or more aggressive local treatment have not changed the predominately local pattern of recurrence, [11–15]. However, other groups have suggested that MGMT promoter methylation [16, 17], CRET [18, 19], intensified local radiotherapy [20] and tumor involvement of the subventricular zone (SVZ) are related with higher rates of distant failure [21].

The relationship between the recurrence location and survival is also complex. While some authors suggest that distant failure is associated with worst overall survival (OS) [22], others have found distant recurrences to be more frequent in cases with longer OS [16].

We retrospectively reviewed our recent series of newly-diagnosed GBM patients treated with 5-ALA guided surgery, and standard radio-chemotherapy with temozolomide, in order to analyse the recurrence pattern and its relationship with different factors.

## Materials and methods

From August 2007, we developed a prospective database on the use of FGS. All patients had given informed consent in accord with the Helsinki convention norms.

From this database, we identified 77 consecutive patients with newly-diagnosed GBM resected. We retrospectively reviewed the pattern of first recurrence after the initial surgery.

The standard management of these patients in our Center had been as follow:

FGS was performed after oral administration of 20 mg/kg of 5-ALA (Gliolan^®^, Medac, Wedel, Germany) 2 h before induction of anesthesia, as published [23]. A Pentero© microscope (Zeiss, Oberkochen, Germany) was used for tumor resection. Volumetric measurement of the tumor volume on T1Gd and T2, specific lobe involvement and eloquent area involvement were analyzed upon preoperative MRI. Tumor volume was measured with manual segmentation using the iplan cranial software (BrainLab). The target of the surgery was the resection of all the fluorescent tissue in the surgery field when it was deemed safe. Neurophysiological monitoring was used for tumors near eloquent areas. When total removal of the fluorescence tissue was not possible, based on monitoring data, it was registered in the surgical sheet. All histological diagnoses were established according to the WHO criteria. MRI images were always obtained within the first 72 h after surgery. Extent of resection (EOR) was calculated as percentage of (preoperative tumor volume-postoperative tumor volume)/preoperative tumor volume [24]. CRET was defined as the absence of contrast enhancement in the volumetric analysis over the T1Gd on postoperative MRI [25]. Follow-up MRIs were done before and after radiotherapy and later every 2–3 months or when the patients experienced neurological deterioration.

Clinical data including age and Karnofsky Performance Score (KPS), and MGMT promoter methylation status as determined by semi-quantitative PCR, were recorded prospectively for every patient. All patients had received standard radio-chemotherapy with temozolomide according to the “Stupp” protocol. Thirty-six cases had also been included in a phase II trial that added autologous dendritic cells vaccination to the standard therapy (EudraCT: 2009-009879-35). After first relapse patients had been treated at the criteria of their neurooncologist, including reoperation and reirradiation if possible, and second-line chemotherapy. The most common salvage therapy consisted on bevazucimab.

For the retrospective review, recurrence was defined according to RANO criteria [26]. Recurrence was classified as local if it was located in the resection cavity or in continuity with it, or less than 2 cm from the primary tumor margins and distant when the recurrence lesion border was more than 2 cm from the previous cavity. For the statistical analysis, the cases were distant and local recurrences appeared at the same time were counted together with the cases with distant only lesion and this group was called non-local. Previous MRI were reviewed to look for abnormal signal in T1Gd or T2 in the area of distant recurrence.

The recurrence location, progression free survival (PFS) and OS data were obtained from the clinical registry in our center. The patients with missing data were contacted by phone if possible.

### Statistical methods

Age and preoperative tumor volume were analyzed as continue variables.

Location of the tumor and recurrences, KPS, MGMT promoter methylation status and CRET were defined as categorical variables.

PFS and OS were analyzed with Kaplan–Meier method; comparisons were made using Log-rank test.

Chi square, *t*-Student tests and Mann–Whitney were used for comparisons in the univariant models, as appropriate.

Multivariate analysis with a logistic regression model was used to assess relationship of age, KPS, MGMT methylation status, CRET, and preoperative volume on recurrence location.

Values with *p* < 0.05 in these analyses were considered statistically significant.

## Results

Out of the 77 patients with first resection for a GBM, 58 have had a documented progression, 10 are alive without recurrence and 9 have died without known progression or we did not had the MRI. Only the 58 patients with documented progression have been included in the analysis.

In 38 out of the 58 patients with a documented progression (65.5 % of the recurrent cases) the recurrent lesion was local, in 16 (27.6 %) was distant, and in 4 cases there were local and distant recurrences at the same time (6.9 %). For comparisons, 34.5 % were classified as non-local progressions, and 65.5 % were only local.

Clinical data of the patients are summarized in Table 1.

**Table 1:** Clinical data of patients progressed

	Local recurrence	Non- local recurrence	*p* value
Mean age	59	58	0.312
T1-Gd preoperative volume	36.7 cc	49.9 cc	<0.001*
T2-FLAIR preoperative volume	92.73 cc	81.75 cc	0.933
Karnofsky			
70–100	72 %	28 %	0.175
<70	54 %	46 %	
MGMT			
Methylated	59 %	41 %	0.107
Non-methylated	68 %	32 %	
Extend of resection			
CRET	59 %	41 %	0.412
No CRET	79 %	21 %	
Included in vaccine trial	46 %	52 %	0.307
Median PFS	6.4	13.8	0.019*
Median OS	19.3	24	0.9

*CRET* complete resection of enhanced tumor, *PFS* progression free survival, *OS* overall survival

CRET was achieved in 44 out of 58 patients; mean EOR in the whole series was 99.27 %. In the 14 cases with some residual enhancing tumor volume, the mean residual enhancing volume was 1.9 cc and the mean EOR was 96.8 %. The proportion of patients with non-local recurrence was greater for cases with CRET (41 vs. 21 %), although the difference did not reach statistical significance (*p* = 0.15, chi-test).

Total resection of fluorescence tissue was achieved in 77 %, and non-local recurrence was greater when all the fluorescence tissue was removed (36.4 vs. 23.1 %), the difference is not statistically significant (*p* = 0.37, chi-test).

MGMT promoter status could not be determined in 8.6 % of the patients (5/58). In the remainder, it was found to be methylated in 42 % and unmethylated in 58 %. The proportion of patients with non-local recurrence was greater for cases with methylated MGMT promoter (41 vs. 32 %), although the difference did not reach statistical significance (*p* = 0.35, chi-test).

Mean preoperative tumor volume measured upon T1Gd was 36.8 cc for patients with local recurrence and 49.9 cc for patients with non-local recurrence (*p* < 0.001 *t* test). Subgroup analysis showed the difference to be due to the fourth quartile: patients with preoperative tumor volumes higher than 54 cc had a 66.6 % rate of non-local recurrence, while the patients within the first, second and third quartiles had rates of 41, 33, and 7 %, respectively.

Mean preoperative volume of the abnormal area in T2 was 87.24 cc. It was 92.73 cc in patients with local recurrence, and 81.75 cc in patients with non-local recurrence (*p* = 0.933).

No signal abnormalities were observed in the area where the distant recurrence later occurred in any case.

Recurrence was non-local in 33.3 % of the patients included in immunotherapy protocol and in 39 % of the patients not included (*p* = 0.42).

**Fig. 1:**
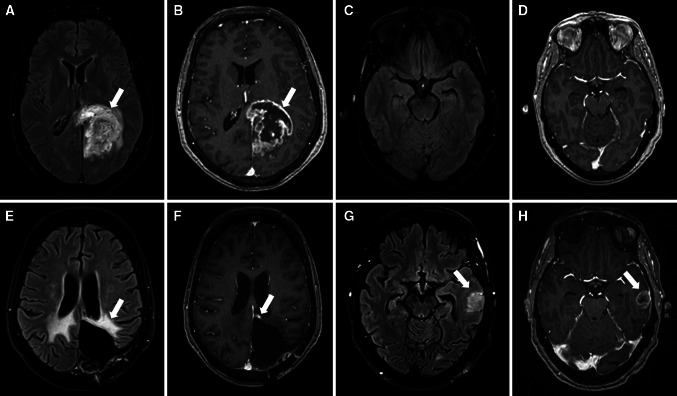
63 years-old female with GBM. MRI at diagnosis (*upper row*) and first recurrence (*lower row*). At diagnosis a left parieto-occipital mass was found, hyperintense in T2 FLAIR (*arrow* in **a**) and with irregular peripheral enhancement with central necrosis in T1Gd (*arrow* in **b**), with normal left temporal lobe (**c** and **d**). 13 months after surgery there was no evidence of recurrence at the surgical cavity borders with only treatment-related changes, with gliosis in T2 FLAIR (*arrow* in **e**) and a small stable area on enhancement in T1 Gad (*arrow* in **f**). Yet a new remote nodule appeared in the left temporal lobe, outside the radiation field (*arrows* in **g** and **h**), confirmed to be a GBM recurrence after surgery

Tumor involving the SVZ was analysed following the classification published before [21], however, in our series a relationship was not found between tumor location and location of recurrence (*p* = 0.6) (Fig. 1).

In the multivariate analysis, the preoperative tumor volume was statistically significant (*p* = 0.014), and CRET was near significance in some models. Both CRET and MGMT promoter had high HR that could suggest influence, albeit they did not reach significance, perhaps due to the small sample size. Table 2 shows the model with CRET, methylation of MGMT promoter, and preoperative volume. The statistical significance did not change in models where we excluded patients with local and distant recurrence from the analysis (data not shown).

**Table 2:** Multivariate analysis including complete resection of enhanced tumor (CRET), fluorescence tissue resection (Fl. Tissue resection), preoperative volume, MEGMT methylation status, age and Karnovsky Performance Score (KPS)

	B	E.T.	Wald	gl	Sig.	Exp (B)
CRET	2.242	1.283	2.743	3.055	1	0.08
Fl. tissue resection	1.366	0.837	2.663	1	0.103	3.92
Preoperative volume	0.032	0.016	3.968	1	**0.046**	1.033
MGMT-met	1.367	0.797	2.946	1	0.086	3.925
Age	−0.014	0.034	0.158	1	0.691	0.986
KPS	0.033	0.031	1.139	1	0.286	1.034

Bold value indicates statistically significant

Median PFS was significantly longer for patients with non-local recurrences than for patients with local recurrences (13.8 vs. 6.4 months; *p* = 0.019, log-rank).

Although OS was different in both groups (24.0 non-local vs. 19.3 local; *p* = 0.9), the difference was not significant, perhaps due to small sample size.

**Fig. 2:**
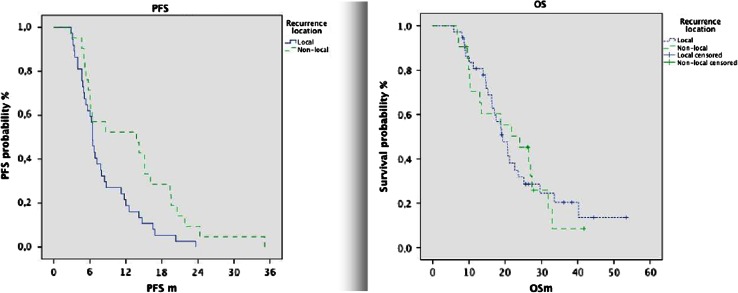
Kaplan–Meier curves for progression free survival (PFS) and overall survival (OS)

Survival since progression was significantly shorter for patients with non-local progression than for patients with local progression (7.5 vs. 12.0 months, *p* = 0.017) (Fig. 2).

Median overall survival of the whole series was 21.2 months.

## Discussion

Non-local recurrence after treatment for glioblastoma have been historically considered very rare. Many authors have reviewed this issue in recent years, after the introduction of radiotherapy and temozolomide as standard, and most of them state that it has not changed, with rates of distant lesions at first recurrence between 2 and 28 %, mostly below 20 % [12–17, 19, 27–30]. However, it has been suggested that some factors increase the rate of distant recurrence only in certain subgroups, such as patients with methylated MGMT promoter [16, 17], very extensive resections [18, 19], and tumor involvement of the subventricular zone (SVZ) [21].

We found a rate of distant first recurrence higher than previous papers. Making comparisons can be difficult, as the patterns of relapse are not universally defined, and different classifications are employed. Usually, the classification used is based on the specific factors being studied. Radiotherapy studies mostly define recurrences in three groups related to the radiation field [12, 16, 27, 28, 30]. Surgery based studies use the distance to the resection cavity [14, 18]. Pope et al. [11, 29] proposed a classification considering both the place and the aspect of the recurrence that have been applied in recent studies of antiangiogenic drugs. Despite the different classifications, two simple groups can be identified in all studies: local recurrence and non-local recurrence. We believe that these two groups summarise the disease behaviour in a simple scheme, making possible to compare results published in the literature. In our series, we found 27.6 % distant only, and 6.9 % distant and local at the same time. The appearance of distant disease marks an important stage in the GBM evolution, with different treatment needs, so the grouping as non-local of all cases with any distant disease makes clinical sense. It is also an easy data to compare between groups. Both the 27.6 % distant only and the 34.5 % non-local are higher than data published before. Table 3 presents the results from the papers that disclose data of the first recurrence in patients who have been treated with the actual standard therapeutic protocol.

**Table 3:** Series giving data about pattern of first recurrence in GBM after radiotherapy plus temozolomide

	Patient number	Local-only recurrences (%)	Non-local recurrences (%)
Series with CRET^a^			
Petrecca K^14^	20	85	15
De Bonis P^18^	75		
Extended resection	27	66.67	33.33
Border resection	48	87.5	12.50
Series with MGMT analysis^b^:			
Brandes AA^16^	79		
Methylated	19	57.87	28.13
Non-methylated	60	85	15
Niyazi M^17^	52		
Methylated	11	64.71	35.29
Non-methylated	16	88.89	11.11
Other series:			
Dobelbower MC^12^	20	80	20
McDonald MW^15^	41	98	2
Milano MT ^28^	47	89	11
Paulsson, AK^13^			
Chamberlain MC^11^	80	80	20

The numbers of patients included in this table are only cases with first recurrence, and the percentage is of the number of first recurrences. Only the data of the patients with known MGMT status has been included

^a^Series that analyze complete resection of enhance tumor (CRET)

^b^Series that give separate information of cases with methylated MGMT promoter versus non-methylated

We analysed our data looking for the factors behind this high rate of non-local first recurrence.

The clinical characteristics of the patients included in the series, based on age and functional status were quite normal and had no correlation to the pattern of recurrence.

MGMT promoter methylation is well recognized as a favourable prognosis factor in GBM and two papers have already reported that the pattern of recurrence can be significantly influenced by it [16, 17]. In these two papers, rate of non-local failure was greater for patients with methylated MGMT promoter, with 42 versus 15 % [16] and 21–8 % [17]. This difference was also present in our series (41 vs 28 %) although the unmethylated group had also a high rate of non-local recurrence, which coupled with the small number of patients, prevented the difference from reaching statistical significance.

The main selection bias of our series is that all the cases were candidates to resection surgery and had extensive removal, even the subgroup with some residual tumor had a mean tumor volume of 1.9 cc. In this regard, is important to point out that the criteria for surgical resection in our department are wide, we exclude for surgery only patients with diffuse invasion of eloquent areas, and most of the patients treated in our institution receive resection surgery.

The intention to do maximal resection is standard in our department, and we have previously shown that the routine use of FGS provides a high rate of CRET and very high mean EOR [6]. Two recent series have focused in the pattern of recurrence in patients with CRET followed by radiotherapy plus temozolomide. Petrecca found only 15 % of non-local recurrences after complete tumor resection and standard treatment with radiotherapy and temozolomide[14]. De Bonis published their data on recurrences after CRET in a very selected population where non-eloquent locations allowed an en bloc resection. This group studied two subsets of patients: one with resection limited to the tumor border (BR) visible in the surgical piece exam and one with extended resection (ER), with more than 1 cm beyond the tumor border. They found a higher than expected rate of non-local recurrences, 33 %, only in the group with ER, with BR having 12.5 %. Our whole series has a percentage of non-local recurrent similar to the group of ER in De Bonis series.

The most likely explanation for the high rate of non-local recurrence is the more extensive resection associated to FGS. The association between greater resection and non-local recurrences has appeared in some previous works only for the most extensive resections. In the present data the rate of non-local relapse is higher for the whole series because FGS guided resections can be more extensive that the resection of the tissue visible in T1Gd MRI. Previous data have shown that the fluorescence induced by 5-ALA show more tumor than the T1Gd sequence [23] [31]. We found than patients with resection deemed complete by both MRI and fluorescent light live longer than those patients with resection complete only by MRI [32]. The tendency of GBM to quick local regrowth is highlighted by reports showing local progression before radiotherapy in 53 [33] and 38 % [34] of patients, probably the elimination of a greater amount of invasive cells around the core of the tumor helps to achieve local control, but do not stop the disease.

We found an influence of preoperative volume that has not been described previously, but offered a robust statistical significance. We added a multivariate analysis that has not been done in previous papers, including all the variables with possible relevance. This model confirms the significance of preoperative tumor volume, and shows CRET as almost significant. We can speculate that the infiltrated brain volume around a big tumor is much bigger than in a smaller tumor due to the exponential relationship between volume and radius.

Treatment after surgery included radiotherapy plus temozolomide as intention to treat in all the patients. The group of patients which received immunotherapy added to the standard had a slightly lower rate of non-local recurrence, so this experimental therapy cannot explain the high rate of non-local recurrence observed, albeit it may have an impact in the OS of the total series.

Lim et al. [21] found an association between distant recurrences and tumor relationship to subventricular zone (SVZ), concluding that those tumors in contact with the SVZ were more likely to have distant recurrences than tumors not involving the SVZ. We did not find this relationship in our series.

PFS was longer in patients with non local recurrences, as reported by other authors [16, 17]. Despite this association, OS difference was small and not reached statistical significance (24.0 non-local vs. 19.3). The better PFS was counterbalanced by a much shorter survival from the time of progression in distant recurrence patients. We believe this paradox indicates how the disease advances. Multicentricity has been associated with poor prognostic in GBM. In the multivariate analysis of the radiographic characteristics made by Pope, multicentricity had the worst OS [22]. The patients with better surgery get more local control and longer PFS but, once a distant lesion appears, the disease has progressed and they have the same short survival from that moment of the initially multicentric, as have been nicely showed previously by Hefti [35].

The PFS and OS data suggest that a very extensive resection can increase the local control, however, the surgery has no effect over distant cells and the cells that infiltrate distally reproduce the tumor. It seems that this cells situated farther and being at low density, need more time to produce a visible recurrence, albeit once the tumor distant recurrence appears, it signals more advanced disease and the remaining survival time is short.

The OS in our series is longer than most series of GBM cases, probably reflecting the combined effect of extended resection with FGS, radiotherapy, temozolomide, plus second line therapies and plus immunotherapy in almost half the patients. Our data suggests than improvement of OS through local control will lead to more distant failure cases, unless we found a therapy to effectively tackle the invasion.

## Conclusions

Our results suggest that improvement in surgical treatment of GBM with FGS added to standard therapy can influence the pattern of relapse, increasing distant recurrences in patients with longer PFS. Greater preoperative enhancing tumor volume is associated with increased rate of non-local recurrences. Novel therapies should be directed to diffuse invasive disease in the brain to be effective.
